# Investigation of Post-Processing of Additively Manufactured Nitinol Smart Springs with Plasma-Electrolytic Polishing

**DOI:** 10.3390/ma14154093

**Published:** 2021-07-22

**Authors:** Vincent N. Stepputat, Henning Zeidler, Daniel Safranchik, Evgeny Strokin, Falko Böttger-Hiller

**Affiliations:** 1 Institute for Machine Elements, Engineering Design and Manufacturing (IMKF), Technische Universität Bergakademie Freiberg, Chair of Additive Manufacturing, Agricolastr. 1, 09599 Freiberg, Germany; vincent.stepputat@imkf.tu-freiberg.de (V.N.S.); DaniS@trdf.technion.ac.il (D.S.); 2 Beckmann-Institut für Technologieentwicklung e. V., Annaberger Str. 73, 09111 Chemnitz, Germany; boettger-hiller@beckmann-institut.de; 3 Technion–Israel Institute of Technology, Technion City, Haifa 3200003, Israel; strokin@trdf.technion.ac.il

**Keywords:** plasma electrolytic polishing, nitinol, laser beam melting, additive manufacturing

## Abstract

Additive manufacturing of Nitinol is a promising field, as it can circumvent the challenges associated with its conventional production processes and unlock unique advantages. However, the accompanying surface features such as powder adhesions, spatters, ballings, or oxide discolorations are undesirable in engineering applications and therefore must be removed. Plasma electrolytic polishing (PeP) might prove to be a suitable finishing process for this purpose, but the effects of post-processing on the mechanical and functional material properties of additively manufactured Nitinol are still largely unresearched. This study seeks to address this issue. The changes on and in the part caused by PeP with processing times between 2 and 20 min are investigated using Nitinol compression springs manufactured by Laser Beam Melting. As a benchmark for the scanning electron microscope images, the differential scanning calorimetry (DSC) measurements, and the mechanical load test cycles, conventionally fabricated Nitinol springs of identical geometry with a medical grade polished surface are used. After 5 min of PeP, a glossy surface free of powder adhesion is achieved, which is increasingly levelled by further polishing. The shape memory properties of the material are retained without a shift in the transformation temperatures being detectable. The decreasing spring rate is primarily attributable to a reduction in the effective wire diameter. Consequently, PeP has proven to be an applicable and effective post-processing method for additively manufactured Nitinol.

## 1. Introduction

As the most widely used shape memory alloy, Nitinol exhibits a combination of characteristic thermomechanical properties that predestine the material for a range of applications. Complementary to excellent mechanical properties and a high resistance to corrosion [1] and wear [2,3], it has outstanding biocompatibility, which qualifies Nitinol for use in load-bearing implants [4,5,6]. However, the basis of today′s widespread application from medical stents to eyeglass frames are the mechanisms of shape memory effect (SME) and superelasticity, which are both based on a phase transformation in the solid state. Nitinol exhibits an ordered BCC lattice structure at high temperatures, which is referred to as austenite. When it is cooled beyond the transformation range, it converts to a less ordered twinned martensite structure. Transformation and re-transformation take place at different temperatures, resulting in a corresponding hysteresis. The characterizing transformation temperatures (TT) of the austenite (Start A_S_, Finish A_F_) and martensite transformation (analogue M_S_, M_F_) as well as the resulting hysteresis width react very sensitively to the chemical composition, in particular the Ni-Ti ratio in the matrix [7]. Another decisive influence is the thermal and mechanical processing involved in the selected production route, whereby each individual step must be considered for a targeted part design in order to achieve the desired functional properties [7,8,9,10,11].

A significant limitation for the industrial use of Nitinol components are the challenges associated with conventional manufacturing processes, especially casting and machining [12,13,14,15,16,17]. Therefore, practically only wrought Nitinol as a wire or band product is used, significantly limiting the achievable geometries. Consequently, additive manufacturing of Nitinol, for example with Laser Beam Melting (LBM), has become a focus of research in recent years, thus enabling the production of complex and even intentionally porous components in one process step [18,19,20,21,22,23,24]. However, the advantages to be realized in this way are accompanied by the need to post-process the surface, primarily to remove powder adhesions and to achieve an application-dependent roughness. While the former may become detached during operation, causing severely increased abrasion or short circuits in voltage bearing parts, the removal of roughness peaks is generally associated with improved mechanical performance. Therefore, especially the surface of dynamically loaded components is predestined for polishing. However, it has been shown that retaining the characteristic fissured surface morphology of additively manufactured parts instead of attempting to level it completely can improve the biocompatibility of Nitinol implants [25]. Plasma electrolytic polishing (PeP), having proven to achieve low roughness and high gloss levels on additively manufactured workpieces within short processing times [26,27,28], shows exceptional potential for this application. The process involves immersing the workpiece in a cathodically contacted electrolyte bath with a material-specific, aqueous salt solution and subsequently contacting it anodically. Providing a potential *u* between 180 and 400 V as well as a current density *J* of approximately 0.2 A∙cm^−2^, a vapor-gaseous envelope forms around the workpiece in which a plasma stabilizes. Here, a combination of electrochemical and physical mechanisms cause the material to be preferentially removed at protruding contours, eliminating burrs and leveling out the surface roughness. While a roughness of Ra < 0.2 µm can be achieved on functional surfaces of milled parts within a few minutes [8], the results are heavily dependent on the initial morphology. Therefore, the significantly higher initial roughness of additively manufactured metal surfaces usually requires longer processing times [29].

To date, the influences of the associated post-processing technologies on the surface shape, the TT, as well as the mechanical and functional properties are largely unresearched. Basics can be found in the investigations of chemical etching of additively manufactured Ni-Ti bone fixation plates by Jahadakbar et al. [30]. These have shown that the process succeeds in a achieving a smoothened, coherent surface free of powder adhesions. However, considerable deviations from the target geometry need to be taken into account, whereby more than 40 wt % of the material must be dissolved again for a smooth-appearing surface. In this context, a reduction of the load-bearing capability could also be observed. However, beyond these specific findings, there is a fundamental lack of insight into the influence of surface finishing technologies on the mechanical and functional properties of additively manufactured Nitinol, especially for the industrially highly relevant electrochemical processes. Such an understanding is necessary to systematically design components along their manufacturing chain and thus qualify the additive manufacturing of Nitinol for industrial use. This paper aims to contribute to this by identifying these key influences for PeP as a highly promising post-processing technology in this area. Potential changes in surface morphology, TT, and mechanical properties during the polishing process are for this purpose investigated on the basis of Nitinol compression springs, which are so-called smart springs. Their function is based on the unconventional mechanical behavior of martensite, which results in a 50% to 75% drop in spring rate compared to austenite [31,32]. At constant load, heating above A_F_ thus leads to a significant reduction in spring deformation. As a result, they react to a change in temperature by performing work and are therefore simultaneously sensors and actuators [33]. Due to their open but non-complex shape, smart springs are suitable for establishing a basis for the evaluation of PeP as a post-processing method for additively manufactured Nitinol components.

## 2. Materials and Methods

**Figure 1:**
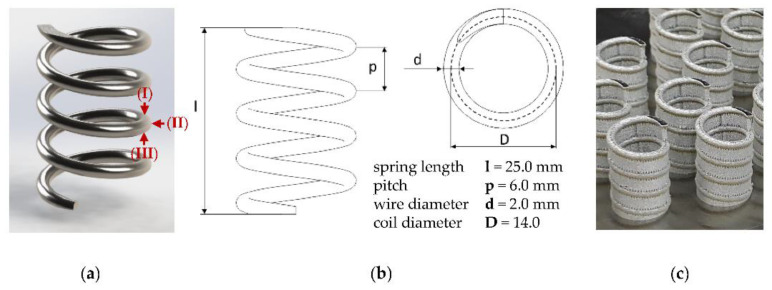
(**a**) CAD rendering of the chosen sample design, indicating the evaluated surface regions: (I) upskin surface, (II) lateral surface, (III) downskin surface, (**b**) spring design parameters, (**c**) as-built samples.

For the purpose of this study, smart springs with a wire diameter (d) of 2 mm, a total length (l) of 25 mm, and ground ends were designed, as given in Figure 1. Thereby, the selected coil diameter ensures that buckling under compressive load in a testing machine is avoided. Following this design, six samples (referred to as A1 to A6) were additively manufactured by LBM on an EOS M 290 (EOS GmbH, Munich, Germany) using Ni_50.8_Ti_49.2_ powder supplied by Sino-Euro Materials Technologies of Xi’an Co. Ltd. (Shaanxi, China) with a particle size distribution of 15–53 µm. The chemical composition of the powder certified by the supplier is given in Table 1. All springs were manufactured in an upright position with identical process parameters (laser power 150 W, laser speed 900 mm∙s^−1^, layer thickness 60 µm, hatch distance 90 µm).

For the evaluation of the polishing results, a comparative benchmark is required, for which the conventional and industry-established production route for nitinol via shape setting was used. The manufacturing of the specimens was carried out by Sino-Euro Materials Technologies of Xi′an Co. Ltd. as well, whereby the alloy composition of the wire shown in Table 1 was selected to ensure that the specimens exhibit preferably identical TT to the LBM powder. According to the chosen design, the cold drawn wires were wound around a mandrel and constrained with the designed pitch. Subsequently, they were heat treated in an inert gas furnace at 500 °C for 20 min and finally cooled down to impress the final shape on the spring. Six samples (referred to as C1 to C6) were manufactured and delivered with a medical-grade polished surface and ground ends, according to the sample design in Figure 1.

**Figure 2:**
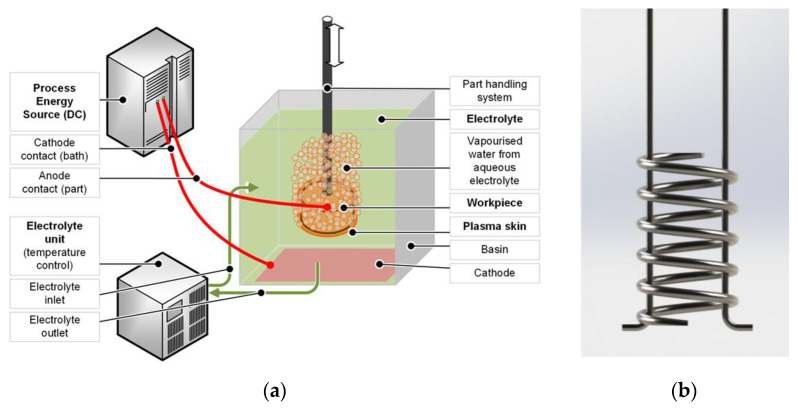
(**a**) Schematic illustration of the experimental PeP setup and (**b**) rendering of the sample holder.

The additively manufactured samples A2 to A6 were PeP processed with different treatment times, as given in Table 2, while A1 remained in the as-built state. As the experiments involved relatively small components and limited quantities, a flexible research setup for PeP was chosen, using an aqueous electrolyte solution. Its effectiveness for the considered material was validated in preliminary tests, whereby roughness values Ra of up to 0.2 µm were achieved on Nitinol wires. For the electrolyte bath, a stainless-steel container filled with 5 L of the electrolyte was selected. Since water evaporates from the boiling electrolyte during the PeP process, the liquid level was checked regularly between the treatments of the individual samples so that it could be refilled if necessary. In this way, the process conditions, especially the immersion depth, were kept constant for all specimens. The complete experimental setup is shown in Figure 2, as well as the sample holder, which was customized to safely guide the workpiece and provide two electrical contacts per spring coil, assuming ideal geometry. This reduces the path of the current through the test specimen and thus the electrical resistance applied by it.

The electrical voltage was set to *u* = 330 V in accordance with previously successful results. Since the removal rate for PeP and thus the achievable roughness on the individual surfaces is known to be dependent on the orientation of the workpiece in the electrolyte bath, uniform processing had to be ensured. For this reason, the springs were kept upright during polishing and turned upside down every 2:30 min. An exception was made for the shortest polishing time of two minutes, for which they were rotated once after one minute.

In order to gain insights into the relationship between the LBM process, PeP parameters, surface characteristics, and thermomechanical properties of the manufactured samples, selected test methods were carried out. A key parameter to determine the performance of an electrolytic polishing process such as PeP is the material removal rate (MRR) in µm per minute. It can be estimated by using the removed mass during the surface treatment process, as difference between initial mass ($m_{0}$) and resulting mass ($m_{1}$), which is related to the PeP time (t), sample density (ρ) and spring surface area (A), as given in Equation (1).

(1) $$MRR=\frac{\left(m_{0}-m_{1}\right)}{t\times \rho \times A}$$

A can be approximated by the surface area of the CAD sample design, which is calculated to be 1153 mm^2^. Assuming that fully dense components have been achieved in the LBM process, a density ρ of 6.45 g·cm^−2^ for Nitinol may be used [34]. Since the polishing time is chosen for the different treatment stages, only the mass of each sample before and after the PeP process remains to be determined. This is carried out using a Kern 572 precision scale (KERN & SOHN GmbH, Balingen-Frommern, Germany) featuring a sample enclosure as well as a measuring resolution of 1 mg.

The changes in surface morphology of the additively manufactured samples through polishing were evaluated after each step on the center coil using a ZEISS Ultra55 (Carl Zeiss Microscopy GmbH, Oberkochen, Germany) scanning electron microscope. Then, the mechanical properties were assessed in a compression test for the A- and C-samples. At a preload of 2 N, each spring was subjected to ten cycles involving a deformation of 5 mm and subsequent unloading, each at 1 mm∙s^−1^ and room temperature (RT). For this purpose, a compression testing machine of type inspekt retrofit AGS-G (Hegewald & Peschke GmbH, Nossen, Germany) was used. The identification of the TT was carried out for samples A1 to A5 using a Mettler-Toledo DSC 3+ differential scanning calorimeter (Mettler-Toledo GmbH, Gießen, Germany), testing the samples in the range of −100 to 100 °C with a temperature rate of 10 K·s^−1^ under nitrogen atmosphere. This required five sections of 4 mm length being cut out from each sample. It is known that plastic deformation leads to residual stresses in the material and thus may skew the results obtained from the thermograph. Therefore, the samples were separated using a diamond cutting disc, while leaving out the spring ends. The specimens were kept free of moisture to avoid artefacts in the analysis and were tested in the DSC shortly after fabrication. In addition to the samples obtained from the additively manufactured springs, the unprocessed LBM powder and the conventionally manufactured sample C-1 were also tested to identify the influence of the manufacturing process on the TT.

## 3. Results and Discussion

### 3.1. Evaluation of the PeP Process Performance

All five additively manufactured smart springs were successfully post-processed with PeP. The MRR characterizes the amount of material removed per unit of time and thus describes the productivity of the polishing process. It is widely used in the field of electrolytic removal processes due to the comparatively slow removal rates. The corresponding material quantity can initially be simply specified in mg. However, in PeP as a surface process, the removal rate is strongly dependent on the surface area of the workpieces. Thus, a calculation in µm·min^−1^ according to Equation (1) leads to more comparable results, as given in Table 3.

**Figure 3:**
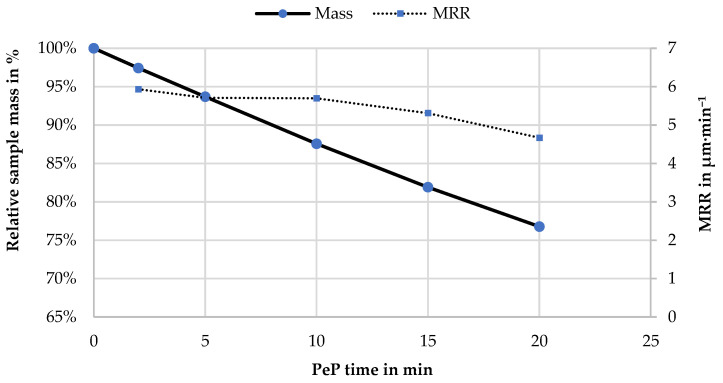
Characterization of the PeP process with increasing processing times by the relative remaining sample mass of sample A6 in percentage and the MRR in µm·min^−1^.

The results are also visualized together with the change in sample mass in Figure 3. It is noteworthy that the mass loss of 1.2% per minute of polishing time for sample 6, which corresponds to about 40 mg, is almost perfectly linear. This indicates stable process conditions. On the other hand, the MRR is relatively constant at 5.7 µm·min^−1^ in the first 10 min and only drops slightly with longer polishing times. In this context, it must be noted that in previous investigations of PeP, a limit of MRR = 5 µm·min^−1^ was determined on the basis of different materials, above which a mirror finish was no longer achieved on the components surface [26]. However, in the present case, the MRR is above 5 µm·min^−1^ over a wide range of the investigated area, and the samples still exhibit a pronounced gloss after 5 min of PeP. Thus, it seems that Nitinol can be processed more quickly than previously assumed, whereby the exact threshold range remains to be determined.

### 3.2. Surface Morphology

**Figure 4:**
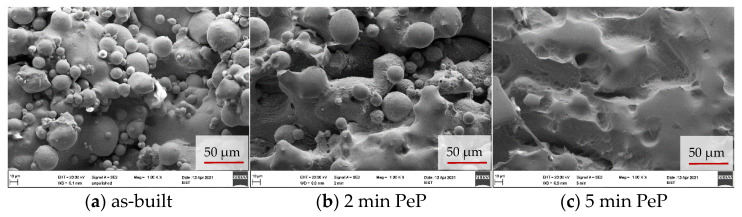
SEM images of the lateral spring surface, revealing the removal of powder adhesions during PeP.

SEM images of the additively manufactured samples clearly reveal the changes in surface morphology caused by PeP, whereas optical microscope images have shown to be impaired by the strong gloss of the samples, appearing after 5 min of polishing. As shown in Figure 4a, the initial state of the springs is characterized by features inherent to LBM, and it exhibits a fissured appearance. Ballings lead to an uneven contour, while adhesions of partially melted powder increase the roughness and pose an operational risk. Removing these, as discussed earlier, is a primary goal of post-processing. It can be seen that 2 min of treatment is not enough, as it requires 5 min of PeP before powder adhesions are no longer visible on the surface. However, at the same time, the fissured morphology of the original state is retained, with protruding contours only being rounded. For this surface condition, an increased biocompatibility compared to flat surfaces could be demonstrated in the literature [25], which indicates that a closer examination of a PeP time of 5 min for load-bearing Nitinol implants is promising.

**Figure 5:**
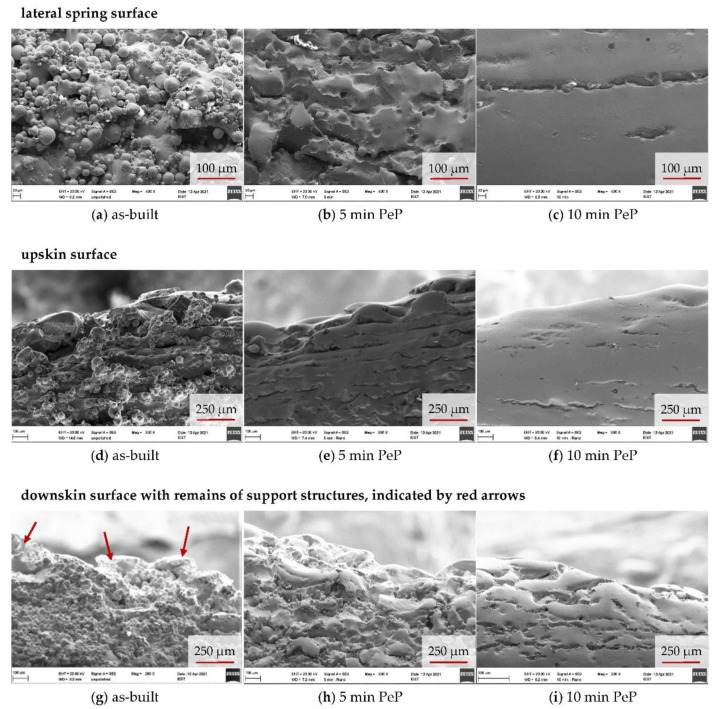
SEM images of detailed surface features on the up- and downskin of the spring: comparison of the as-built LBM sample to the springs after 5 min and 10 min of PeP.

With longer polishing and thus further material removal, the pronounced hills and valleys are reduced, resulting in a coherent, considerably smoother appearing surface after 10 min. However, as Figure 5 shows, there are distinct differences between the surfaces in relation to their orientation to the build direction of the spring. The lateral areas appear smooth and feature only isolated fissures. It is noteworthy that these occur with regular spacing and always run parallel to the build plane of the LBM process. This implies that they are located at boundaries between layers with incomplete bonding around the edges. A review of Figure 5d–f reinforces this conclusion. The upskin surface of the springs shown here was on top during LBM manufacturing and is therefore geometrically defined by stacked ovals corresponding to the slicing of the component. The absence of layer boundaries causes less fissures and a more continuous appearance of the surface.

**Figure 6:**
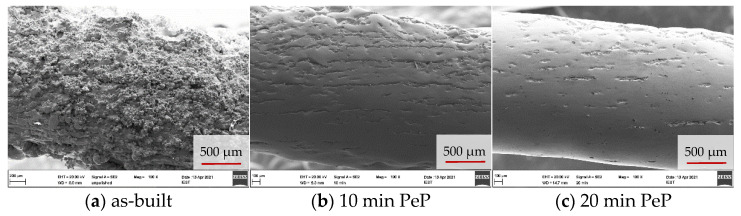
SEM overview images of the lateral spring surface after different PeP times.

A similar pattern could be expected on the opposing downskin surface. However, the support structures connected here have left behind corresponding sharp-edged and clearly protruding burrs after manual removal with pliers. In this respect, much more material has to be removed by PeP in order to achieve a smooth, continuous appearance. Since the material is eroded evenly, processing the underside of the springs requires considerably more time. This is manifested in the fact that its appearance after 10 min corresponds most to the lateral and upskin surface after 5 min and thus requires twice the PeP time for the same results. As shown in Figure 6, longer polishing times of more than 10 min result in a less significant improvement of the appearance of the lateral and downskin surfaces compared to the first minutes of PeP. While the remains of the support structures could be completely leveled after 20 min, the fissures along the build layers are still visible, albeit reduced. If required, these could be removed by even longer processing, but at the expense of further material loss. However, understanding that the polishing result in PeP strongly depends on the initial state of the surface, it seems more promising to counteract the occurrence of the fissures by optimizing the LBM process. Furthermore, support structures should be thoroughly removed in order to achieve a homogeneous processing result on the entire component.

### 3.3. Phase Transformation Response

The DSC measurements in the temperature range of −100 to 100 °C could be carried out successfully. The TT values determined from the thermographs are summarized in Table 4, whereby the hysteresis width is calculated as the difference between A_F_ and M_S_.

**Figure 7:**
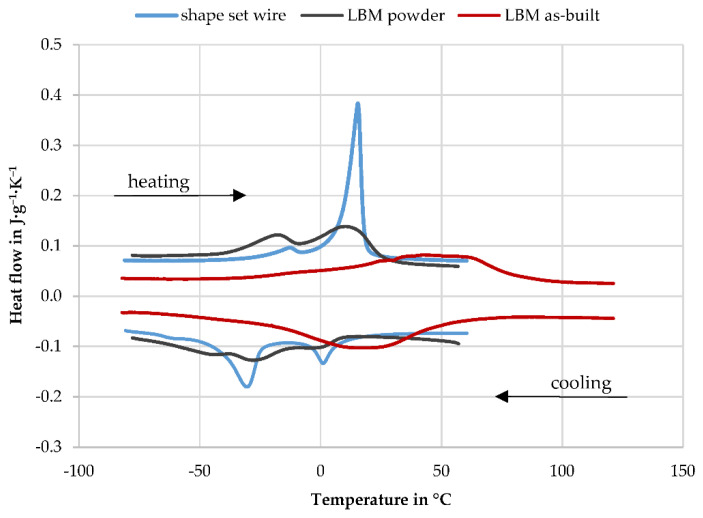
DSC thermograph of the shape-set wire, the unprocessed LBM powder, and the as-built LBM sample.

In Figure 7, it can be seen that the objective of selecting the nitinol alloy to match the TT of the powder has been fulfilled. Chemical analyses are available for both, showing a nickel content of 50.93 at % for the shape set springs and 51.51 at % for the LBM powder; see Table 1. The transformation temperatures respond very sensitively to this, with M_S_ dropping by about 93 °C per at % nickel above 50 at % [7]. This would initially suggest that both samples have a difference of 54 K in their transformation temperatures. However, this is not the case. Instead, the peaks of the latent transformation heats occur at approximately the same temperature but are significantly less pronounced for the powder. The flatter and less distinctive transformation peaks result in a smaller slope of the aligned tangents and thus more extreme transformation temperatures, whereas the beginning and the end of the observable transformation are almost congruent. In this respect, the thermomechanical processing during manufacturing and the resulting microstructure compensated for the difference in chemical composition. However, the LBM process introduces a significant change in the material, whereby the selected process parameters play a decisive role. A clear increase in the transformation temperatures with a pronounced widening of the hysteresis can be observed in the case of A_F_ by 65.5 K to 91.0 °C and in the case of M_S_ by 58.4 K to 50.6 °C. This is most likely due to the evaporation of nickel from the melt pool caused by the high temperatures in the build process [8,10,35]. The shape set wire is always in the austenitic state at RT (ϑ = 21 °C), as all transformations take place below this point. However, the resulting transformation hysteresis for the additively manufactured springs shows that the dominant phase at RT depends on their thermal history. If the sample originated from the cold state (ϑ < −23 °C), the transformation into austenite just started at RT, and the material is thus nearly fully martensitic. If it instead cooled down from high temperatures (ϑ > 91 °C), the martensitic transformation started at 50.6 °C. At RT, a certain proportion of the phase is martensitic and the rest austenitic. Since samples A1 to A6 originated from the LBM process and were not subjected to temperatures below RT, the latter is the case. This behaviour is of decisive importance for further investigations.

**Figure 8:**
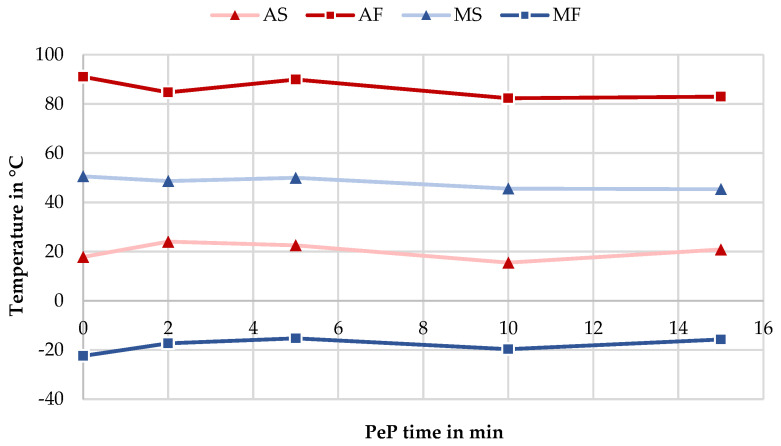
Change of TT with increasing PeP time, obtained by DSC measurements on each sample.

For the LBM smart springs, as visualized in Figure 8, the graphical plot of TT over polishing time only shows fluctuations of a few K, but no discernible trends. Therefore, it can be concluded that the PeP process does not influence the transformation behavior of additively manufactured nitinol components. The electrochemical removal mechanism could possibly cause slight shifts in the Ni-Ti ratio in the matrix of the surface layer, but these if they exist at all evidently do not play a significant role, even with the large surface-to-volume ratio of the given springs.

### 3.4. Mechanical Properties

**Figure 9:**
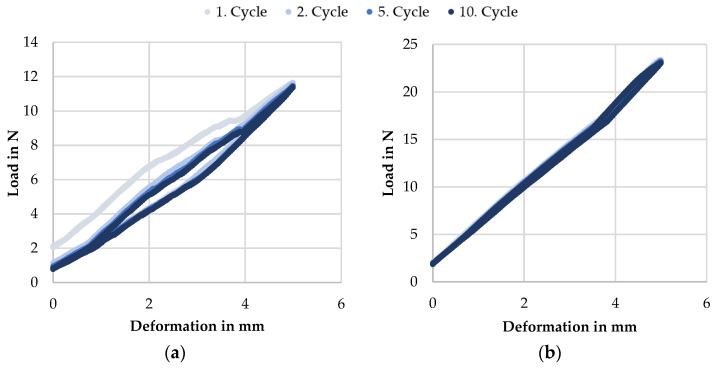
Compression behavior of the smart springs during load–deformation cycles exemplified on the basis of cycles 1, 2, 5, and 10 for (**a**) additively manufactured sample A3 (5 min PeP) and (**b**) conventionally shape set sample C1.

The compression tests of the additively manufactured samples at RT reveal a similar behavior for A1 to A6, as exemplified in Figure 9a by sample A3. Starting with a preload of 2 N, the spring settles at the first deformation and begins the second cycle at about 1 N in each case. The loss of spring force ranges between 0.81 and 1.14 N with a mean value of 1.0 N and no discernible correlation to the PeP process. However, after the first setting, both the start and end points of the load–deformation cycle remain constant in the further stages. From the third to the tenth cycle, the curves are congruent and therefore can be used for a reliable determination of the mechanical properties. Furthermore, a deviation between the deformation and recovery curves can be seen, which is referred to as a hysteresis. Due to the design, the material or the force application, and dissipation points, frictional forces have a deformation- and recovery-inhibiting effect. Since the area under the spring curve corresponds to the spring work, this can be understood as the lost work or damping of the spring. Its extent, expressed by the width of the hysteresis, shows no discernible differences between the samples A1 to A6.

A different result is obtained with the conventionally manufactured specimens. As shown in Figure 9b for sample C1, there is no discernible loss of force between the cycles, and the hysteresis is also much less pronounced. Since the outer shape and clamping conditions were identical for all of them during testing, the reason for the difference must be found in the material itself. Here, it is reasonable to assume that the characteristic microstructure of an additively manufactured component in technical use leads to higher internal friction than that of a drawn wire. From the lack of influence of PeP on the damping behavior, it can also be concluded that no narrow hysteresis can be achieved on additively manufactured springs by this polishing process alone. However, with regard to the influence of the microstructure, it is likely that an optimization can be realized by an adapted choice of process parameters for LBM.

Based on the tenth and last cycle, the spring rate as a practically relevant mechanical property for the design of smart springs can be determined. For this purpose, only the deformation curve was considered for each sample to identify its slope by means of linear regression. The approximation proved to be of high quality, with the coefficient of determination R^2^ always exceeding 0.996. The results are given in Table 5.

With a standard deviation of 0.38 N∙mm^−1^, the spring rates for the C samples show a comparatively high dispersion around the mean value of 3.98 N∙mm^−1^. Neither the thermal history nor the test conditions differed between the specimens. The former would also be insignificant, since the shape set springs are always austenitic at RT, as the previously discussed DSC results demonstrate. In addition to the random distribution, geometric deviations in the production process, which influence the spring deflection behavior, could also be considered to explain the scatter. It has to be taken into account that the ends were not manufactured as closed and ground. A detailed examination of the curves shows that the slope changes slightly at different points, e.g., in Figure 9b at 3.6 mm deformation. If, with increasing compression of the spring, two coils come into contact, the number of free coils is reduced, and the spring rate increases. If this point is shifted in the measuring range due to deviations in manufacturing, it explains the scatter of the calculated spring rates. This explanation is congruent with the recorded curves.

A comparison of the additively manufactured springs to the samples produced by the shape setting shows that the spring rate of the latter is about twice as high. Since this applies to all polishing conditions, the cause cannot be found in the material removal. The number of coils is also identical; thus, only the shear modulus G remains as an influencing variable on the spring rate. It can be concluded that the shear modulus for the additively manufactured springs is about half the value to be expected for conventional components at RT. As concluded above, the latter have an austenitic crystal structure here. The microstructure of the springs produced with LBM, on the other hand, is likely to have already partially transformed into martensite with the temperature falling below M_S_ at 50.6 °C during cooling after the build process. This transformation extends to −22.4 °C and thus over a temperature range of 73 °C, of which only the first 29 °C were reached at RT. Thus, the springs are still expected to be dominantly austenitic with minor amounts of martensite. However, these reduce the shear modulus due to the differences in the mechanical properties of both crystal structures. In addition, the observed difference could also be caused by a lower stiffness as a result of impurities and porosity originating from the additive manufacturing process. Both factors cannot be clearly distinguished on the basis of the conducted investigations.

**Figure 10:**
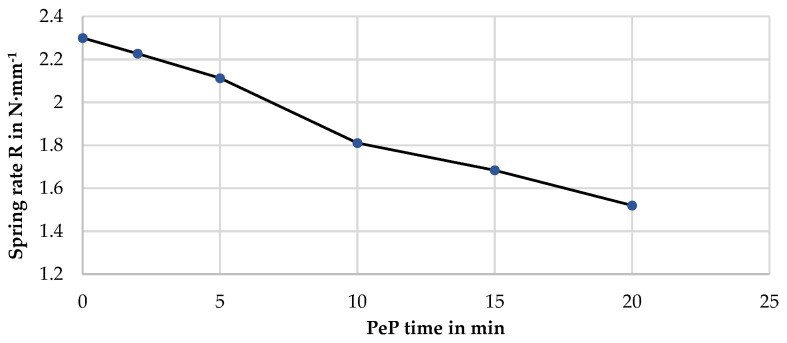
Change of spring rate under compression with increasing PeP time, obtained by linear regression from the loading curve of the 10th cycle with R^2^ > 0.996 for each sample.

If the spring rate of the additively manufactures specimens is plotted against the corresponding polishing time, the curve shown in Figure 10 is obtained.

As expected, a decrease in spring rate can be observed, which develops almost linearly over the polishing time. Due to the progressive removal of material, the effective wire diameter is reduced, which in turn contributes to the fourth power in the calculation of the spring rate [36]. As a result, after only 10 min and thus at the time when a smooth-appearing surface is achieved on the side and bottom surfaces of the spring, the work capacity is already reduced by 22%. Therefore, in the design of additively manufactured Nitinol components whose surface is intended to be post-processed with PeP, a corresponding allowance must be provided. This can be compared with the results obtained from the MRR model. Assuming an MRR of 5.7 µm·min^−1^ as determined in Section 3.1, the wire diameter is reduced by 228 µm after 20 min. This corresponds to 11.4%. Since d contributes to the fourth power, the new spring rate should account for about 61.6% of the initial value. The compression tests provide a value of 66.1%. This shows that the MRR model provides a satisfactory approximation for the material removal during PeP for additively manufactured nitinol specimens as well. Although an ideal cylinder was assumed for the purpose of this model, the uniformity of the interacting mechanisms leads to realistic assumptions about the effects of PeP on the functional properties.

## 4. Conclusions

Based on a shared design, Nitinol smart springs were manufactured both by LBM and by conventional shape setting. In the as-built condition, the additively manufactured samples exhibited a rough surface characterized by ballings and powder adhesions. These features are undesirable in engineering applications and must therefore be removed during post-processing. With the help of PeP, the smart springs were successfully polished, with the SEM images revealing that after 5 min of processing, no more powder adhesions were visible. Prolonged polishing levels the fissured structures characteristic of LBM at about 40 mg/min, resulting in a smoother appearing, coherent surface. However, it features occasional fissures at the boundaries of adjacent layers, the formation of which should be counteracted by optimizing the LBM process. While satisfactory polishing results were achieved on the lateral and upper surfaces, the removal of the support structure remains a challenge. Thus, the result on the downskin surfaces fell short of the rest. Therefore, it can be concluded that support structures should be thoroughly removed mechanically prior to PeP. The DSC measurements demonstrated that the LBM manufacturing process causes a significant upward shift of the TT by approximately 60 K, which must be considered during part design. However, no indications for a further influence of PeP could be found. In the compression test, which was repeated in ten cycles, clear differences were observed between the additively and conventionally manufactured springs. The former show a significantly more pronounced damping characteristic and a force loss of about 1 N before the behavior levels off after the third cycle. Both of these features are hardly or not at all detectable in the load–deformation curves of the shape set springs. However, they exhibit a roughly twice as high shear modulus. This is presumably due to the presence of martensite in the matrix of the LBM springs at RT, impairing the mechanical properties. Understanding these differences in compression behavior motivates further investigations of additively manufactured and PeP-polished Nitinol. In addition, extensive studies on the effects of PeP on biocompatibility and corrosion behavior are planned in order to provide reliable findings on its applicability in the medical industry.

## Figures and Tables

**Table 1:** Chemical composition of the LBM powder and shape set wire as certified by the supplier in wt %, requirements according to ASTM F-2063.

Element	Ni	Fe	C	O + N	Nb	Cu	Cr	H	Ti
Specification	54.5–57.0	<0.050	<0.050	<0.050	<0.025	<0.010	<0.010	<0.005	Bal.
LBM powder	56.570	0.021	0.009	0.043	<0.010	<0.010	<0.010	0.002	Bal.
Shape set wire	56.000	0.060	0.004	0.042	0.001	0.002	0.009	0.001	Bal.

**Table 2:** Overview of PeP treatment times for each sample.

Sample No.	A1	A2	A3	A4	A5	A6
polishing time	0 min	2 min	5 min	10 min	15 min	20 min

**Table 3:** Overview over the PeP treatment times for each sample.

Sample No.	A1	A2	A3	A4	A5	A6
polishing time in min	0 min	2 min	5 min	10 min	15 min	20 min
MRR in µm·min^−1^	-	5.94	5.71	5.70	5.31	4.67

**Table 4:** DSC measurement results for tested samples.

Sample No.	C1	Powder	A1	A2	A3	A4	A5
polishing time	-	-	0 min	2 min	5 min	10 min	15 min
A_S_	8.3 °C	−12.6 °C	17.8 °C	24.0 °C	22.5 °C	15.5 °C	20.8 °C
A_F_	18.0 °C	25.5 °C	91.0 °C	84.7 °C	89.9 °C	82.3 °C	82.9 °C
hysteresis width	42.7 K	33.3 K	40.4 K	36.1 K	49.9 K	36.7 K	37.5 K
M_S_	−24.7 °C	−7.8 °C	50.6 °C	48.6 °C	50.0 °C	45.6 °C	45.4 °C
M_F_	−41.0 °C	−45.00 °C	−22.4 °C	−17.3 °C	−15.3 °C	−19.7 °C	−15.8 °C

**Table 5:** Calculated spring rates R for all measured samples.

**Sample No.**	**A1**	**A2**	**A3**	**A4**	**A5**	**A6**
Polishing time	0 min	2 min	5 min	10 min	15 min	20 min
R in N∙mm^−1^	2.30	2.23	2.11	1.81	1.68	1.52
**Sample No.**	**C1**	**C2**	**C3**	**C4**	**C5**	**C6**
R in N∙mm^−1^	4.23	4.00	3.65	3.35	4.18	4.50

## Data Availability

Data sharing is not applicable for this article.
